# Use of nebulized liposomal amphotericin B and posaconazole as antifungal prophylaxis in patients with severe SARS-CoV2 infection in intensive care unit

**DOI:** 10.1007/s15010-024-02234-9

**Published:** 2024-03-26

**Authors:** Benedetta Fumarola, Liana Signorini, Silvia Lorenzotti, Paola Lanza, Barbara Saccani, Evelyn Van Hauwermeiren, Alice Mulè, Simone Piva, Matteo Rota, Francesco Zuccalà, Francesco Antonio Rasulo, Matteo Filippini, Alberto Bertazzoli, Giovanni Del Fabro, Alberto Matteelli

**Affiliations:** 1 Clinic of Infectious Diseases, ASST Spedali Civili, Brescia, Italy; 2 Department of Clinical and Experimental Medicine, University of Brescia, Brescia, Italy; 3 Department of Medical and Surgical Specialties, Radiological Science and Public Health, University of Brescia, Brescia, Italy; 4 Department of Anesthesia, Critical Care and Emergency, Spedali Civili University Hospital, Brescia, Italy; 5 Department of Molecular and Translational Medicine, Università degli Studi di Brescia, Brescia, Italy; 6 Department of Anesthesia and Intensive Care, Spedali Civili Hospital, Brescia, Italy; 7 Intensive Care and Anesthesiology, Department of Surgical Specialties, Radiological Sciences and Public Health, University of Brescia and Spedali Civili Hospital, Brescia, Italy; 8 Department of Infectious Diseases, ASFO “Santa Maria Degli Angeli” Hospital of Pordenone, Pordenone, Italy

**Keywords:** CAPA, COVID-19, Antifungal prophylaxis, Intensive care unit

## Abstract

**Purpose:**

COVID-19 associated pulmonary aspergillosis (CAPA) is common and linked with high fatality rates. To assess the impact on the incidence and outcome of CAPA of an antifungal prophylaxis (AFP) we compared two cohorts of COVID-19 patients admitted to intensive care units (ICU) in Brescia, Italy, from January to August 2021.

**Methods:**

The study cohort included all mechanically ventilated patients observed between April 2021 and August 2021 with SARS-CoV-2-pneumonia, who received AFP with oral posaconazole (200 mg every 6 h) and nebulized liposomal amphotericin B (50 mg every 2 weeks) from ICU admission to 7 days after discharge or, if applicable, until tracheostomy removal. The control cohort included COVID-19 patients admitted to the same ICU between January and March 2021 who did not receive any AFP. Subjects with CAPA at ICU admission were excluded.

**Results:**

We included 270 patients, of whom 64 (23.7%) received AFP. In patients in the study group, CAPA-related mortality was significantly reduced (29% vs. 48% p = 0.04), as well as the incidence of CAPA (3.1% vs 12.1%, p = 0.03). Patients who developed CAPA were older (mean of 70-y-old vs 63-y-old, p < 0.001). One subject discontinued posaconazole due to an adverse reaction. Among the 46 patients who received it, only one patient reached an effective plasma concentration of posaconazole.

**Conclusion:**

AFP was associated with reduced incidence and mortality from CAPA and was well tolerated in patients with severe COVID-19. Posaconazole concentrations below the efficacy threshold in almost all patients may be attributable to drug interactions and prompt further studies to define its clinical significance.

## Introduction

*Aspergillus spp.* is a ubiquitous saprophytic mold commonly found in soil, water and building materials, capable of causing a wide range of lung diseases, ranging from simple hypersensitivity reactions to an invasive disease with high fatality [1]. Among the different manifestations, Invasive Pulmonary Aspergillosis (IPA) is the most severe clinical picture, and usually occurs in patients who are immunocompromised, those suffering of a prolonged neutropenia and those undergoing hematopoietic cell transplantation or on prolonged therapy with glucocorticoids or immunosuppressive drugs [2]. Histologically, it is characterized by the invasion of lung tissue by the hyphae of *Aspergillus spp*., which can also penetrate inside the pulmonary vessels and arterioles and cause micro ischemic events, pulmonary necrosis, and dissemination to other organs [3, 4]. Viral coinfections can cause direct damage to the epithelium of the respiratory tract and can lead to local and/or systemic dysregulation of the immune system, facilitating bacterial and fungal superinfections [5, 6]. Recent cohort studies have reported influenza-associated pulmonary aspergillosis (IAPA) as a severe secondary infection with poor outcome [7–11]. During the SARS-CoV-2 pandemic, many cases of COVID-19 associated pulmonary aspergillosis (CAPA) were reported [12–16]. In a prospective cohort of 108 severely compromised patients with ARDS, the 30-day mortality was higher in patients with CAPA than in those without it (44% versus 19%) [17]. The diagnosis of CAPA is usually difficult because *Aspergillus spp.* is a frequent colonizer of the airways, the invasive form lacks a typical radiological picture, and it can occur in the absence of evident host immune depression [18, 19].

Several studies have shown that prophylactic therapy with azoles and liposomal amphotericin B (AmB) can reduce the incidence of invasive pulmonary aspergillosis and the resulting mortality in neutropenic or immunocompromised hematological patients [20–23]. The American Society of Clinical Oncology and Infectious Disease Society of America (IDSA) Clinical Practice Guidelines Update recommends prophylaxis with mold-active oral triazole (posaconazole, voriconazole, and isavuconazole) or a parenteral echinocandin in patients experiencing extended periods of neutropenia and at > 6% risk for IPA [24]. A literature search, mainly based on observational studies, performed by Duckwall et al. suggested that inhaled liposomal amphotericin B could be a valid alternative for invasive aspergillosis prophylaxis in high-risk neutropenic patients with hematologic malignancies and stem cell transplant recipient [22]. During the COVID-19 pandemic Van Ackerbroeck et al. and Soriano et al. described their positive experiences with the use of nebulized liposomal amphotericin B as aspergillosis prophylaxis: they administered 12.5 mg and 50 mg of inhaled liposomal amphotericin B twice weekly, respectively, and observed a significant reduction in the incidence of CAPA in ventilated COVID-19 patients [25, 26]. Despite the potential local side effects associated with the use of nebulized amphotericin B, such as dyspnea, cough, and bronchospasm, it is associated with reduced systemic toxicity and fewer drug-drug interactions than the systemic formulation [22].

At the beginning of 2021, we faced an outbreak of pulmonary aspergillosis in patients with severe SARS-CoV-2 infection admitted to intensive care unit (ICU). As a response, we set up a prophylactic intervention with inhaled liposomal amphotericin B and posaconazole oral suspension (or micafungin in those on posaconazole-interacting therapy).

We here report the impact of antifungal prophylaxis on the incidence of CAPA and its associated mortality in ICU-admitted patients with SARS-CoV-2 infection.

## Methods

### Study design, location, and population

We present the results of a single-center, prospective intervention aimed at reducing the incidence of COVID-19 Associated Pulmonary Aspergillosis (CAPA) in patients admitted to the ICU at Spedali Civili, Brescia, Italy, spanning from January 1, 2021, to January 31, 2022. During the study's early months until the conclusion of March 2021, adults with confirmed SARS-CoV-2 infection via positive polymerase chain reaction (PCR) on nasopharyngeal swab (NPS), who required invasive mechanical ventilation at Intensive Care Units 1 and 2 of ASST Spedali Civili, Brescia, did not receive antifungal prophylaxis (referred to as the NoPROPH cohort).  Subsequently, commencing from April 2021, all patients with COVID-19 admitted to the ICU and necessitating invasive mechanical ventilation, devoid of clinical, radiological, and microbiological signs of CAPA received antifungal prophylaxis (referred to as the PROPH cohort). Patients meeting any of the following criteria were excluded from the study:  (1) age < 18 years; (2) admitted to the ICU for reasons other than COVID-19 and with incidental PCR finding for SARS-CoV-2 positive on NPS; (3) CAPA occurring within 72 h of ICU stay.

### Diagnosis of CAPA

The diagnosis of CAPA was established following the guidelines outlined by the European Confederation of Medical Mycology (ECMM)/International Society of Human and Animal Mycoses (ISHAM) consensus of 2020. Indirect microbiological diagnosis was based on the titer of galactomannan (GM) in broncho-alveolar lavage (BAL) samples. Until May 2021, the Platelia test was employed, with a recognized positivity threshold of  > 0.5. Subsequently, from May 2021 onwards, the laboratory shifted towards the Virclia test, a chemiluminescence semiautomatic immunoassay with a positivity cut-off set at ≥ 0.2 for both BAL and serum samples.

### Prophylactic scheme

**Fig. 1:**
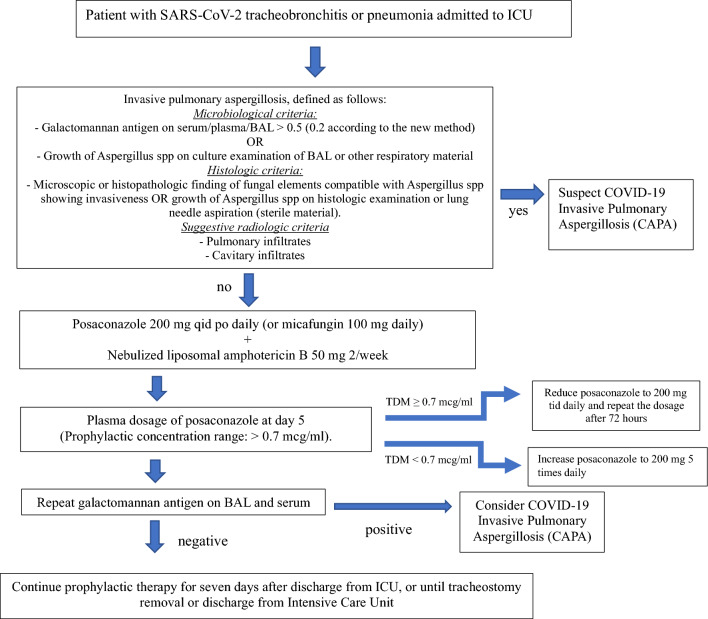
Flow-chart of management and treatment steps in patients included in the experimental arm. *TDM* therapeutic drug monitoring, *BAL* bronchoalveolar lavage

The prophylactic scheme is shown in Fig. 1. Posaconazole (POS) oral suspension at a dosage of 200 mg four times daily (QID) (or micafungin 100 mg every 24 h in case of drug interactions between posaconazole and the patient’s therapy) and nebulized liposomal amphotericin B were used. Specifically, 50 mg of Ambisome^®^ (Gilead Sciences, Inc.), dissolved in 10 ml of sterile water, was nebulized twice weekly through ventilation tubes.

As part of standard of care, all patients entering ICU due to COVID-19 received methylprednisolone at a dosage of 1 mg/kg for a minimum of 10.

Prophylaxis was sustained for seven days following discharge from the ICU or until tracheostomy removal if present. If invasive pulmonary aspergillosis requiring treatment occurred, prophylaxis was discontinued and treatment with intravenous liposomal amphotericin B was initiated.

### Collected data

Data regarding sex, age, comorbidities, length of stay, type of prophylaxis used, and liposomal amphotericin B consumption were retrospectively collected and analyzed to evaluate potential differences between the two patient cohorts.

Following the initiation of prophylaxis, plasma levels of posaconazole were measured on day 5/7. The threshold plasma target concentration of posaconazole to be achieved was > 700 ng/ml.

Galactomannan antigen in both serum and BAL was monitored once a week until discharge from the intensive care unit.

### Data management

Laboratory, clinical, and radiologic data were retrieved from our in-house electronic health database system and stored in a predefined electronic reporting form (eCRF) using REDCap electronic data capture. The extraction of liposomal amphotericin B consumption was conducted via the pharmacy management program of ASST Spedali Civili Brescia.

### Statistical analysis

Quantitative variables were characterized using either mean and standard deviation (SD) or median and interquartile range (IQR), as deemed appropriate. Categorical variables were summarized as counts and percentages. Comparison between the PROPH group and the NoPROPH group and between the group that developed CAPA and the group without CAPA for all variables of interest was performed using the Wilcoxon-Mann Whitney U test for quantitative variables and the chi-square test, with p values calculated by Monte Carlo simulation (B = 2000). The association between CAPA development and prophylaxis was assessed using both uni- and multi-variate logistic regression models. Results are reported as odds ratio (OR) and 95% confidence interval (CI). A Cox model with time-dependent variable was used to determine the development of CAPA over time.

### Ethics

This study was approved by the Ethical Board of Brescia Province and conducted according to the Declaration of Helsinki and to principles of Good Clinical Practice (GCP). As this study had a retrospective component and was based on routinely collected data, patients’ informed consent was not required according to the Italian law (Italian Guidelines for classification and conduction of observational studies, established by the Italian Drug Agency, “Agenzia Italiana del Farmaco – AIFA” on March 20, 2008). Moreover, for this study we used the general authorization of the Italian Guarantor for the use of retrospective demographical and clinical data, which have been anonymized and treated according to Italian current laws.

## Results

### Sample characteristics

Between January 1, 2021, and January 31, 2022, a total of 293 patients were admitted to the ICU due to COVID-19. Among them, 23 patients were excluded from the analysis as they were diagnosed with CAPA within 3 days of ICU admission. Of the remaining 270 patients, 70% (188/270) were male, with a mean age of 64.9 years (SD 12.1), and 53% were aged over 65 years. Hypertension (57%), heart disease (23%) and dyslipidemia were the most common comorbidities. A small proportion of patients (3.7%) had hematological disease.

**Fig. 2:**
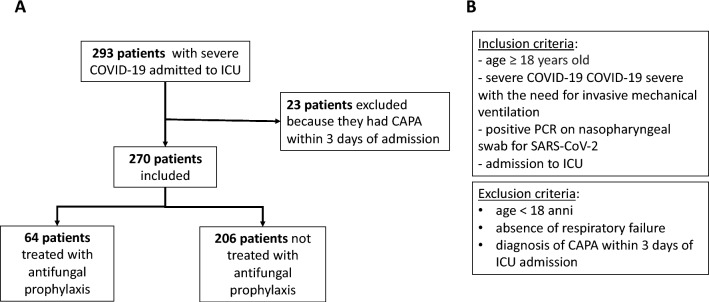
Flow chart of patients enrolled in the study. **A** Initially, 293 patients were enrolled. 23 patients were excluded because they entered ICU already with CAPA (positive galactomannan antigen finding within 3 days of ICU entry). Therefore, 270 patients were included in the analyses, of whom 64 received antifungal prophylaxis and 206 did not. **B** Study inclusion and exclusion criteria. *COVID-19* coronavirus 19 disease, *CAPA* COVID-19 Associated Pulmonary Aspergillosis, *SARS-CoV-2* coronavirus severe acute respiratory syndrome 2, *ICU* intensive care unit

In the study cohort, 64 patients (23.7%) received antifungal prophylaxis (PROPH Group) and 206 (76.3%) did not (NoPROPH Group) (Fig. 2). Demographic, clinical and hospitalization characteristics were similar between the two groups, as shown in Table 1. The 64 treated patients received liposomal aerosolized amphotericin B in combination with posaconazole (AmB + POS; 61, 95.3%) or micafungin (AmB + MIC; 3, 4.7%).

**Table 1:** Baseline characteristics of the study population-distribution stratified on the basis of taking or not taking antifungal prophylaxis

Variable	Total (n = 270)^1^	No antifungal prophylaxis (n = 206)^1^	Antifungal prophylaxis (n = 64)^1^	p value^2^
Demographic variables				
Age (years), average	64.2 (12.06)	64.9 (12.5)	61.9 (10.1)	0.08
Male sex	188 (70)	143 (69)	45 (70)	0.9
Comorbidity				
Heart disease	61 (23)	51 (25)	10 (16)	0.13
Arterial hypertension	153 (57)	120 (58)	33 (52)	0.3
Dyslipidemia	55 (20)	45 (22)	10 (16)	0.3
Hepatopathy	2 (0.7)	2 (1)	0 (0)	> 0.9
Diabetes mellitus	56 (21)	44 (21)	12 (19)	> 0.7
Chronic dialysis	3 (1.1)	3 (1.5)	0 (0)	> 0.9
Neurological disorders	20 (7.4)	16 (7.8)	4 (6,2)	0.8
Primary immunodeficiency	1 (0.4)	1 (0.5)	0 (0)	> 0.9
CRI without the need for dialysis	11 (4.1)	8 (3.9)	3 (4.7)	0.7
Autoimmune diseases	11 (4.1)	7 (3.4)	4 (6.2)	0.3
Hematologic diseases	10 (3.7)	9 (4.4)	1 (1.6)	0.5
Pneumopathies	32 (12)	27 (13)	5 (7.8)	0.3
Tumors	25 (9.3)	22 (11)	3 (4.7)	0.15
Antifungal prophylaxis				
Time from admission to ICU to start of prophylaxis (days)	NA	NA	2.1 (6.6)	NA
Antifungal prophylaxis	NA	NA		NA
- Posaconazole + nebulized liposomal amphotericin B			− 61 (95.3)	
- Micafungin + nebulized liposomal amphotericin B			− 3 (4.7)	
Specific drugs				
Corticosteroids	270 (100)	206 (100)	64 (100)	NA
Outcome				
CAPA	64 (23.7)	25 (12.1)	2 (3.1)	**0.03**
Death	84 (31)	64 (31)	20 (31)	> 0.9
Length of hospitalization in ICU, days	15.3 (13.1)	14.3 (11.4)	18.5 (17.3)	0.07
Length of hospitalization, days	25.6 (19.2)	24.4 (18.8)	29.5 (20.3)	0.08

Bold value indicate significance of p value (p < 0.05)

*CRI* chronic renal failure, *NA* not applicable

^1^Variable are expressed as number (%) and mean (SD)

^2^Pearson’s Chi-squared test; Welch Two Sample t-test; Fisher’s exact test

The overall mean length of hospitalization was 25.6 days (SD 19.24), with an avarage of  15.3 (SD 13.1) spent in the ICU. Patients in the PROPH group had a mean hospital stay of 29.5 days (SD 20.3), including 18.5 days (SD 17.3) in the ICU, whereas patients in the NoPROPH group had a mean hospital stay of 24.4 days (SD 18.8), with 14.3 days (SD 11.3) spent in the ICU. All patients admitted to the ICU received systemic glucocorticoids.

### Incidence of CAPA

Overall, 27 incident cases of CAPA were identified, with 25 cases (92.6%) occurring in the NoPROPH group and 2 in the PROPH group. The incidence of CAPA was significantly higher in the NoPROPH group [25/206 (12.1%) vs 2/64 (3.1%), p = 0.03]. On average, subjects with CAPA were older (mean age 70 (SD 6.44) vs 63.5 (SD 12.43), p < 0.001). The mean time from admission to the ICU to the development of CAPA was 9 days (IQR 7—16), with only one case occurring after more than 30 days (56 days). Prophylaxis for the PROPH group was started on average 2.1 days (SD 6.564) after ICU admission and had a mean duration of 26.8 days (SD 46.8). On the patients with CAPA, thirteen (48%) out of 27 died, compared to 21 out of 243 patients without CAPA (29%) (p = 0.04). Both the length of hospital stay (37.67 vs 24.26 days, p = 0.005) and ICU stay (22.41 vs 14.53, p < 0.001) were significantly shorter in patients without CAPA (Table 2).

**Table 2:** Baseline characteristics of the study population-distribution stratified on the basis of developing CAPA or not

Variables	Total (n = 270)^1^	No CAPA (n = 243)^1^	CAPA (n = 27)^1^	p value^2^
Demographic variables				
Age (years), average	64.2 (12.1)	63.5 (12.4)	70.1 (6.4)	** < 0.001**
Male sex	188 (70)	167 (69)	21 (78)	0.3
Comorbidity				
Heart disease	61 (23)	54 (22)	7 (26)	0.7
Arterial hypertension	153 (57)	133 (55)	20 (74)	**0.05**
Dyslipidemia	55 (20)	50 (21)	5 (19)	0.8
Hepatopathy	2 (0.7)	1 (0.4)	1 (3.7)	0.2
Diabetes mellitus	56 (21)	51 (21)	5 (19)	0.8
Chronic dialysis	3 (1.1)	3 (1.2)	0 (0)	> 0.9
Neurological disorders	20 (7.4)	20 (8.2)	0 (0)	0.2
Primary immunodeficiency	1 (0.4)	1 (0.4)	0 (0)	> 0.9
CRI without the need for dialysis	11 (4.1)	9 (3.7)	2 (7.4)	0.3
Autoimmune diseases	11 (4.1)	11 (4.5)	0 (0)	0.6
Hematologic diseases	10 (3.7)	9 (3.7)	1 (3.7)	> 0.9
Pneumopathies	32 (12)	26 (11)	6 (22)	0.11
Tumors	25 (9.3)	19 (7.8)	6 (22)	**0.03**
Antifungal prophylaxis				
Duration of prophylaxis	26.8 (46.8)	13 (5.7)	27.3 (47.5)	0.65
Time from start of ICU entry to start of prophylaxis, days	2.1 (6.6)	0 (0)	2.1 (6.6)	0.52
Outcome				
Death	84 (31)	71 (29)	13 (48)	**0.04**
Length of hospitalization in ICU, days	15.3 (13.1)	14.5 (13.2)	22.4 (10.5)	** < 0.001**
Duration of total hospitalization, days	25.6 (19.2)	24.3 (18.5)	37.7 (22)	**0.005**

Bold values indicate significance of p value (p < 0.05)

*SD* standard deviation, *CRI* chronic renal failure

^1^ Variables are expressed as numbers (%) and means (SD)

^2^Pearson’s Chi-squared test; Welch Two Sample t-test; Fisher’s exact test

**Fig. 3:**
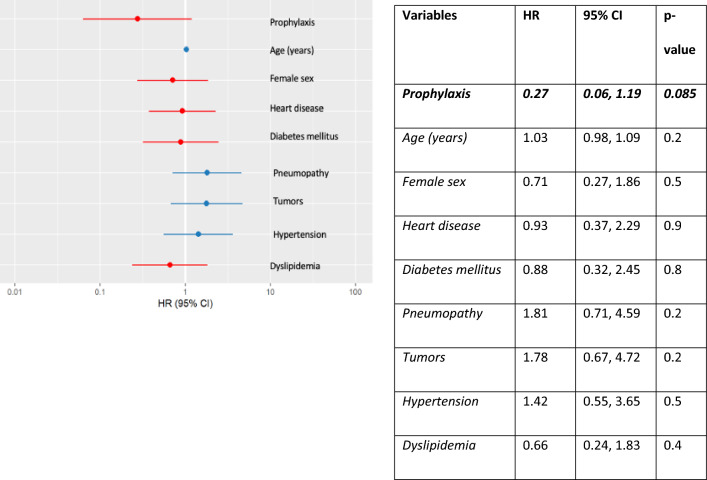
Multivariate COX regression model for the development of CAPA adjusted for prophylaxis, age, sex, heart disease, diabetes mellitus, pulmonary disease, cancer, hypertension, and dyslipidemia. *HR* Hazard Ratio, *CI* Confidence Interval

To evaluate the impact of antifungal prophylaxis on the development of CAPA, we first conduct a univariate regression analysis, revealing that antifungal prophylaxis significantly reduced the incidence of CAPA (HR 0.23, CI 95% 0.05–0.99, p = 0.049). A multivariate regression analysis, adjusted for prognostic co-variates including age, sex, heart disease, diabetes mellitus, pulmonary disease, tumors, hypertension, and dyslipidemia, confirmed this result (HR 0.27, CI 95% 0.06–1.19, p = 0.085), albeit with borderline significance, possibly influenced by a small sample size effect (Fig. 3).

### TDM of posaconazole

**Fig. 4:**
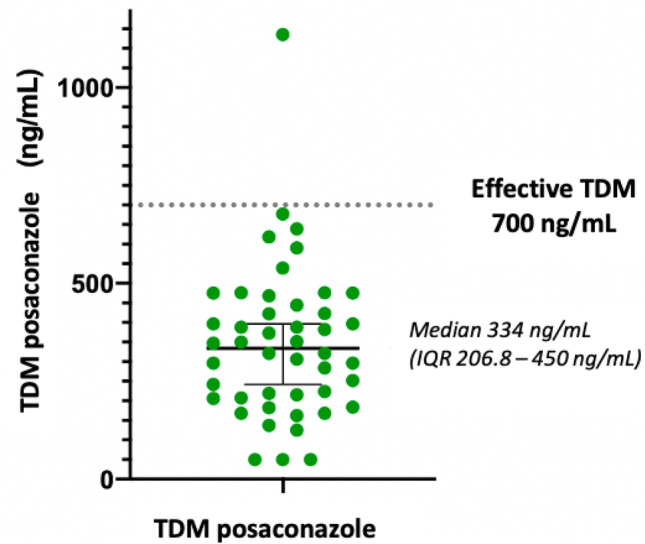
Graphic representation of blood concentrations of posaconazole in the study population. *TDM* therapeutic drug monitoring

Among the 61 patients who received posaconazole prophylaxis, 46 (75%) underwent measurement of plasma concentrations 5–7 days after the first drug administration. The median posaconazole concentration was 0.334 mg/L (IQR 0.207–0.450 mg/L), with the highest recorded value being 1.135 mg/L, and the lowest value falling below 0.05 mg/L (Fig. 4). Only one patient (2.2%) achieved effective plasma concentrations. One subject discontinued posaconazole treatment due to the development of hypertransaminasemia and he carried on with amphotericin B liposomal aerosol alone.

## Discussion

The awareness that fungal infections may complicate viral respiratory diseases has only recently emerged. One of the largest clinical trials, spanning over a seven-year period and involving more than 400 ICU patients, showed that influenza served as an independent risk factor for the development of IAPA and was associated with an increased mortality rate [8–10].

Our study, focusing on patients admitted to the ICU for severe COVID-19, suggests that antifungal prophylaxis may effectively reduce the incidence of CAPA compared to no intervention, (HR 0.27, CI 95% 0.06–1.19, p = 0.085). Furthermore, our data confirmed that COVID patients with CAPA co-infection exhibits increased mortality rates (48% vs 29%, p = 0.04) and prolonged lengths of hospitalization in both the ICU (22.41 days vs 14.53 days, p < 0.001) and overall length of hospitalization (37.67 days vs 24.26 days, p = 0.005) compared to those without CAPA.

Our study was prompted by the increase in the incidence of CAPA in COVID patients in the ICU following February 2021. This rise was likely attributable to renovation works conducted during that period in the ICU wards. It is well-established that *Aspergillus* spores are released in large quantities during construction and renovation works, resulting in increased colonization among hospitalized patients and instances of invasive pulmonary aspergillosis [27]. In response, in April 2021 we introduced systematic fungal prophylaxis, leading to a noticeable reversal in the CAPA incidence trend. Our regimen consisted in a combination of systemic posaconazole (or micafungin) with nebulized liposomal amphotericin B, aimed at reducing fungal load in the lower airways and consequently mitigating the risk of CAPA development.

The choice of prophylactic regimen used in our Center was based on several considerations derived from analysis of the data and recommendations reported in the literature. Posaconazole is the prophylactic azole of choice according to several guidelines [28, 29]. Its effectiveness is demonstrated by two clinical trials conducted using posaconazole as an oral suspension [20, 21]. Hatzl et al. published the results of their experience on case management of CAPA [30]; they treated 75/132 patients with antifungal prophylaxis (98% received intravenous posaconazole) and observed 10 cases of CAPA, including nine in the group without prophylaxis and one in the group with prophylaxis [incidence of 1.4% (95% CI 0.2–9.7) in the PROPH group and 17.5% (95% CI 9.6–31.4) in the NoPROPH group; p = 0.002]. Despite this, they observed no difference in 30-day mortality [30].

To date, there are two ongoing randomized controlled trials on the use of either posaconazole (NCT05065658) or isavuconazole (NCT04707703) for CAPA prevention in critically ill subjects, the results of which are not yet available.

Due to interactions between triazoles and newer oncologic agents, short-term prophylaxis regimens increasingly adopt echinocandins. However, infections have been frequently observed during such prophylaxis [31–33]. In a small subset of patients, we replaced posaconazole with micafungin based on studies demonstrating its superior efficacy compared to echinocandines in preventing invasive fungal infections among neutropenic hematology patients [31–33].

Regarding the use of nebulized liposomal amphotericin B, its efficacy has been evaluated in only a few clinical trials, none of which compare its prophylactic efficacy with that of other anti-mold drugs. A recent review, mostly based on observational studies, showed promising outcomes regarding the use of aerosolized amphotericin B as prophylaxis for invasive pulmonary aspergillosis in hematologic and immunocompromised patients undergoing therapy for allogeneic stem cell transplantation, particularly with the liposomal formulation at a dosage of 12.5 mg twice weekly. As described by others, we too observed that nebulized liposomal amphotericin B may occasionally elicit local adverse effects such as dyspnea, bronchospasm, and coughing [22]. Filters were regularly cleaned and descaled to avoid flow obstruction.

Van Ackerbroeck et al. administered 12.5 mg of inhaled liposomal amphotericin B twice weekly and reported a significant reduction in the incidence of CAPA among ventilated COVID-19 patients [25]. Soriano et al., in response to the experience of their Belgian colleagues and an outbreak of invasive pulmonary aspergillosis in ICU, likely linked to recent renovations, began administering 50 mg of nebulized liposomal amphotericin B every 48 h to critically ill patients, alongside implementing a series of cleaning measures in the rooms undergoing renovation [26]. Despite the persistence of *Aspergillus* spp. contamination in the air for 34 consecutive days, none of the patients who received nebulized liposomal amphotericin B developed CAPA. The only two patients who developed invasive fungal infection had discontinued prophylaxis due to the appearance of bronchospasm [26].

To date no published study has yet evaluated the efficacy of combining two molecules for prophylaxis. We opted for a combination of two drugs because we speculated that the burden of bronchoalveolar colonizers would be abated by nebulized amphotericin B, and that this action would be complemented by the effect on the blood of systemic posaconazole for the few hyphae entering circulation.

Given the wide interindividual and intraindividual variations in the bioavailability and drug interactions of posaconazole, we conducted therapeutic drug monitoring (TDM) to ensure adequate exposure to the drug. Most experts suggest maintaining a plasma concentration above 0.7 mg/l, and the guidelines for TDM of posaconazole prophylaxis currently recommend target levels of 0.7 mg/l after 7 days of treatment [34, 35]. The rationale is based on a Food and Drug Administration analysis of pharmacokinetic data from studies by Ullmann et al. [21] and Cornely et al. [20], who observed that clinical failure with these posaconazole values was 25 percent, with minimal improvement at higher concentrations [34]. Posaconazole is available in three formulations with different pharmacodynamic and pharmacokinetic properties: intravenous formulation, delayed-release tablets, and oral suspension. In our study, we utilized the oral suspension for patients treatment, as it is more easily administered via nasogastric tube, given that the intravenous formulation is unavailable in Italy. Our pharmacokinetic measurements showed that one single patients among 46 (2.2%) reached target levels, with median plasma concentrations being 0.334 mg/L. Mian et al. also observed similar results in their experience [36].

We speculate that this observation is likely attributable to various factors, including the characteristics of the patients admitted to the ICU, the drug administration modality, and drug interactions. Posaconazole absorption in oral suspension is fourfold higher when administered with a high-fat meal compared with a fasting state. In this regard, several studies have shown sub-therapeutic posaconazole concentrations in patients with no or limited food intake, after administration via nasogastric tube, or in patients treated with proton pump inhibitors, H2-antagonists, or metoclopramide, all of which are present in our population. In addition, the pharmacokinetics of the drug under critical conditions may vary greatly due to several physiological factors, such as hypoalbuminemia, as posaconazole binds strongly and in a high percentage (> 98%) to proteins, especially to serum albumin [37].

Our study has limitations. First, mainly, the retrospective observational design, and the absence of standardized diagnostic methods. The change of the galactomannan detection test in May 2021 jeopardized the diagnosis of CAPA; Virclia test, burdened by a higher number of false positives results compared with Platelia, uses cut-off values that are not endorsed by guidelines or the literature. The cut-offs are simply established by the Company. Moreover, the use of non-validated and non-standardized nebulizers for aerosolization of liposomal amphotericin B solution also makes it difficult to compare the different studies, although the use of jet machines should limit the confounding effect [22].

### Conclusion

The increased mortality and length of hospital stay of CAPA patients admitted to ICU may justify the use of antifungal prophylaxis, especially in settings where there is an increased risk of colonization by *Aspergillus*  spp., such as during renovation or construction works. Our data suggest that aerosolized liposomal amphotericin B, administered at a dosage of 50 mg twice weekly, combined with a program of cleaning and maintenance of the filters used to nebulize it, represents an effective and well tolerated approach to mitigate the incidence of CAPA among severely ill patients admitted to the ICU for severe COVID-19. In contrast, the use of posaconazole oral suspension, the first choice in neutropenic hematologic patients, may not be adequate in this population due to several drug interactions and reduced gastrointestinal absorption in ICU patients, which prevent the achievement of effective serum concentrations. Micafungin at the prophylactic dosage of 100 mg every 24 h combined with aerosolized liposomal amphotericin B could be an alternative to posaconazole oral suspension, in view of the fewer drug interactions.

## Data Availability

The data are available and will be provided upon a reasonable request. Requests should be addressed to the corresponding author, along with the reason for seeking access to the data.

## References

[1] Kanj A, Abdallah N, Soubani AO. The spectrum of pulmonary aspergillosis. Respir Med. 2018. 10.1016/j.rmed.2018.06.029. 30053957 10.1016/j.rmed.2018.06.029

[2] Manuel RJ, Kibbler CC. The epidemiology and prevention of invasive aspergillosis. J Hosp Infect. 1998. 10.1016/S0195-6701(98)90323-1. 9651854 10.1016/S0195-6701(98)90323-1

[3] Chamilos G, Kontoyiannis DP. Aspergillus, Candida, and other opportunistic mold infections of the lung. In: Fishman’s Pulmonary Diseases and Disorders, 5th Edition; 2015.

[4] Dagenais TRT, Keller NP. Pathogenesis of Aspergillus fumigatus in invasive aspergillosis. Clin Microbiol Rev. 2009. 10.1128/CMR.00055-08. 19597008 10.1128/CMR.00055-08 PMC2708386

[5] Short KR, Kasper J, Van Der Aa S, et al. Influenza virus damages the alveolar barrier by disrupting epithelial cell tight junctions. Eur Respir J. 2016. 10.1183/13993003.01282-2015. 26743480 10.1183/13993003.01282-2015

[6] Herold S, Becker C, Ridge KM, Budinger GRS. Influenza virus-induced lung injury: Pathogenesis and implications for treatment. Eur Respir J. 2015. 10.1183/09031936.00186214. 25792631 10.1183/09031936.00186214

[7] Van De Veerdonk FL, Kolwijck E, Lestrade PPA, et al. Influenza-associated aspergillosis in critically ill patients. Am J Respir Crit Care Med. 2017. 10.1164/rccm.201612-2540LE. 28387526 10.1164/rccm.201612-2540LE

[8] Schauwvlieghe AFAD, Rijnders BJA, Philips N, et al. Invasive aspergillosis in patients admitted to the intensive care unit with severe influenza: a retrospective cohort study. Lancet Respir Med. 2018. 10.1016/S2213-2600(18)30274-1. 30076119 10.1016/S2213-2600(18)30274-1

[9] Chen L, Han X, Li Y, Zhang C, Xing X. Invasive pulmonary aspergillosis in immunocompetent patients hospitalised with influenza A-related pneumonia: a multicenter retrospective study. BMC Pulm Med. 2020. 10.1186/s12890-020-01257-w. 32907585 10.1186/s12890-020-01257-w PMC7479745

[10] Ku YH, Chan KS, Yang CC, Tan CK, Chuang YC, Yu WL. Higher mortality of severe influenza patients with probable aspergillosis than those with and without other coinfections. J Formos Med Assoc. 2017. 10.1016/j.jfma.2017.06.002. 28647219 10.1016/j.jfma.2017.06.002

[11] Schwartz IS, Friedman DZP, Zapernick L, et al. High rates of influenza-associated invasive pulmonary aspergillosis may not be universal: A retrospective cohort study from alberta, canada. Clin Infect Dis. 2020. 10.1093/cid/ciaa007. 31905235 10.1093/cid/ciaa007

[12] Koehler P, Cornely OA, Böttiger BW, et al. COVID-19 associated pulmonary aspergillosis. Mycoses. 2020. 10.1111/myc.13096. 32339350 10.1111/myc.13096 PMC7267243

[13] Alanio A, Dellière S, Fodil S, Bretagne S, Mégarbane B. Prevalence of putative invasive pulmonary aspergillosis in critically ill patients with COVID-19. Lancet Respir Med. 2020. 10.1016/S2213-2600(20)30237-X. 32445626 10.1016/S2213-2600(20)30237-X PMC7239617

[14] van Arkel ALE, Rijpstra TA, Belderbos HNA, van Wijngaarden P, Verweij PE, Bentvelsen RG. COVID-19-associated pulmonary aspergillosis. Am J Respir Crit Care Med. 2020. 10.1164/rccm.202004-1038LE. 32396381 10.1164/rccm.202004-1038LE PMC7328331

[15] Nasir N, Farooqi J, Mahmood SF, Jabeen K. COVID-19-associated pulmonary aspergillosis (CAPA) in patients admitted with severe COVID-19 pneumonia: an observational study from Pakistan. Mycoses. 2020. 10.1111/myc.13135. 32585069 10.1111/myc.13135 PMC7361517

[16] Arastehfar A, Carvalho A, van de Veerdonk FL, et al. COVID-19 associated pulmonary aspergillosis (CAPA)—from immunology to treatment. J Fungi. 2020. 10.3390/jof6020091. 10.3390/jof6020091 PMC7346000 32599813

[17] Bartoletti M, Pascale R, Cricca M, et al. Epidemiology of invasive pulmonary aspergillosis among intubated patients with COVID-19: a prospective study. Clin Infect Dis. 2020. 10.1093/cid/ciaa1065. 32719848 10.1093/cid/ciaa1065 PMC7454393

[18] Gangneux JP, Reizine F, Guegan H, et al. Is the covid-19 pandemic a good time to include aspergillus molecular detection to categorize aspergillosis in icu patients? A monocentric experience. J Fungi. 2020. 10.3390/jof6030105. 10.3390/jof6030105 PMC7558333 32664423

[19] Koehler P, Bassetti M, Chakrabarti A, et al. Defining and managing COVID-19-associated pulmonary aspergillosis: the 2020 ECMM/ISHAM consensus criteria for research and clinical guidance. Lancet Infect Dis. 2021. 10.1016/S1473-3099(20)30847-1. 33333012 10.1016/S1473-3099(20)30847-1 PMC7833078

[20] Cornely OA, Maertens J, Winston DJ, et al. Posaconazole vs. fluconazole or itraconazole prophylaxis in patients with neutropenia. N Engl J Med. 2007. 10.1056/nejmoa061094. 17251531 10.1056/nejmoa061094

[21] Ullmann AJ, Lipton JH, Vesole DH, et al. Posaconazole or fluconazole for prophylaxis in severe graft-versus-host disease. N Engl J Med. 2007. 10.1056/nejmoa061098. 17251530 10.1056/nejmoa061098

[22] Duckwall MJ, Gales MA, Gales BJ. Inhaled amphotericin B as aspergillosis prophylaxis in hematologic disease: an update. Microbiol Insights. 2019. 10.1177/1178636119869937. 31496719 10.1177/1178636119869937 PMC6716174

[23] Ullmann AJ, Aguado JM, Arikan-Akdagli S, et al. Diagnosis and management of Aspergillus diseases: executive summary of the 2017 ESCMID-ECMM-ERS guideline. Clin Microbiol Infect. 2018. 10.1016/j.cmi.2018.01.002. 29544767 10.1016/j.cmi.2018.01.002

[24] Taplitz RA, Kennedy EB, Bow EJ, et al. Antimicrobial prophylaxis for adult patients with cancer-related immunosuppression: ASCO and IDSA clinical practice guideline update. J Clin Oncol. 2018. 10.1200/JCO.18.00374. 30179565 10.1200/JCO.18.00374

[25] Van Ackerbroeck S, Rutsaert L, Roelant E, Dillen K, Wauters J, Van Regenmortel N. Inhaled liposomal amphotericin-B as a prophylactic treatment for COVID-19-associated pulmonary aspergillosis/aspergillus tracheobronchitis. Crit Care. 2021. 10.1186/s13054-021-03728-w. 34412686 10.1186/s13054-021-03728-w PMC8374123

[26] Soriano MC, Narváez-Chávez G, López-Olivencia M, Fortún J, de Pablo R. Inhaled amphotericin B lipid complex for prophylaxis against COVID-19-associated invasive pulmonary aspergillosis. Intensive Care Med. 2022. 10.1007/s00134-021-06603-y. 34940907 10.1007/s00134-021-06603-y PMC8697542

[27] Talento AF, Fitzgerald M, Redington B, O’Sullivan N, Fenelon L, Rogers TR. Prevention of healthcare-associated invasive aspergillosis during hospital construction/renovation works. J Hosp Infect. 2019;103:1–12. 10.1016/j.jhin.2018.12.020. 30629998 10.1016/j.jhin.2018.12.020

[28] Teh BW, Yeoh DK, Haeusler AA, Committee GS, et al. Consensus guidelines for antifungal prophylaxis in haematological malignancy and haemopoietic stem cell transplantation, 2021. Intern Med J. 2021;51:67–88. 10.1111/imj.15588. 34937140 10.1111/imj.15588

[29] Maertens JA, Girmenia C, Brüggemann RJ et al. European Conference on Infections in Leukaemia (ECIL), a joint venture of the European Group for Blood and Marrow Transplantation (EBMT), the European Organization for Research and Treatment of Cancer (EORTC), the Immunocompromised Host Society (ICHS) and, & European Conference on Infections in Leukaemia (ECIL), a joint venture of the European Group for Blood and Marrow Transplantation (EBMT), the European Organization for Research and Treatment of Cancer (EORTC), the Immunocompromised Host Society (ICHS) and the European LeukemiaNet (ELN) (2018). European guidelines for primary antifungal prophylaxis in adult haematology patients: summary of the updated recommendations from the European Conference on Infections in Leukaemia. J Antimicrob Chemother. 2018;73:3221–3230. 10.1093/jac/dky286 10.1093/jac/dky286 30085172

[30] Hatzl S, Reisinger AC, Posch F, et al. Antifungal prophylaxis for prevention of COVID-19-associated pulmonary aspergillosis in critically ill patients: an observational study. Crit Care. 2021. 10.1186/s13054-021-03753-9. 34526087 10.1186/s13054-021-03753-9 PMC8441945

[31] Jenks JD, Cornely OA, Chen SCA, Thompson GR, Hoenigl M. Breakthrough invasive fungal infections: Who is at risk? Mycoses. 2020. 10.1111/myc.13148. 32744334 10.1111/myc.13148

[32] Van Burik JAH, Ratanatharathorn V, Stepan DE, et al. Micafungin versus fluconazole for prophylaxis against invasive fungal infections during neutropenia in patients undergoing hematopoietic stem cell transplantation. Clin Infect Dis. 2004. 10.1086/422312. 15546073 10.1086/422312

[33] Madureira A, Bergeron A, Lacroix C, et al. Breakthrough invasive aspergillosis in allogeneic haematopoietic stem cell transplant recipients treated with caspofungin. Int J Antimicrob Agents. 2007. 10.1016/j.ijantimicag.2007.07.026. 18029149 10.1016/j.ijantimicag.2007.07.026

[34] Jang SH, Colangelo PM, Gobburu JVS. Exposure-response of posaconazole used for prophylaxis against invasive fungal infections: evaluating the need to adjust doses based on drug concentrations in plasma. Clin Pharmacol Ther. 2010. 10.1038/clpt.2010.64. 20505665 10.1038/clpt.2010.64

[35] Dekkers BGJ, Bakker M, van der Elst KCM, et al. Therapeutic drug monitoring of posaconazole: an update. Curr Fungal Infect Rep. 2016. 10.1007/s12281-016-0255-4. 27358662 10.1007/s12281-016-0255-4 PMC4896980

[36] Mian P, Trof RJ, Beishuizen A, Masselink JB, Cornet AD, Sportel ET. Suboptimal plasma concentrations with posaconazole suspension as prophylaxis in critically ill COVID-19 patients at risk of Covid-associated pulmonary aspergillosis. J Clin Pharm Ther. 2022. 10.1111/jcpt.13518. 34431552 10.1111/jcpt.13518 PMC9528909

[37] Summary of Product Characteristics – Noxafil 40 mg/mL oral suspension (posaconazole). MSD. Accessed via https://www.ema.europa.eu/en/documents/product-information/noxafil-epar-product-information_en.pdf (**date of revision of the text January 2022**)

